# Use of a Feed-Forward Back Propagation Network for the Prediction of Small for Gestational Age Newborns in a Cohort of Pregnant Patients with Thrombophilia

**DOI:** 10.3390/diagnostics12041009

**Published:** 2022-04-16

**Authors:** Petronela Vicoveanu, Ingrid Andrada Vasilache, Ioana Sadiye Scripcariu, Dragos Nemescu, Alexandru Carauleanu, Dragos Vicoveanu, Ana Roxana Covali, Catalina Filip, Demetra Socolov

**Affiliations:** 1 Department of Obstetrics and Gynecology, ‘Grigore T. Popa’ University of Medicine and Pharmacy, 700115 Iasi, Romania; petronelapintilie@yahoo.com (P.V.); isscripcariu@gmail.com (I.S.S.); dnemescu@yahoo.com (D.N.); acarauleanu@yahoo.com (A.C.); demetrasocolov@gmail.com (D.S.); 2 Faculty of Electrical Engineering and Computer Science, Stefan cel Mare University of Suceava, 720229 Suceava, Romania; dragos.vicoveanu@usm.ro; 3 Department of Radiology, Elena Doamna Obsterics and Gynecology University Hospital, 700398 Iasi, Romania; rcovali@yahoo.com; 4 Department of Vascular Surgery, ‘Grigore T. Popa’ University of Medicine and Pharmacy, 700115 Iasi, Romania; filipcatalina20@gmail.com

**Keywords:** machine learning, thrombophilia, small for gestational age

## Abstract

(1) Background: Fetal growth restriction is a relatively common disorder in pregnant patients with thrombophilia. New artificial intelligence algorithms are a promising option for the prediction of adverse obstetrical outcomes. The aim of this study was to evaluate the predictive performance of a Feed-Forward Back Propagation Network (FFBPN) for the prediction of small for gestational age (SGA) newborns in a cohort of pregnant patients with thrombophilia. (2) Methods: This observational retrospective study included all pregnancies in women with thrombophilia who attended two tertiary maternity hospitals in Romania between January 2013 and December 2020. Bivariate associations of SGA and each predictor variable were evaluated. Clinical and paraclinical predictors were further included in a FFBPN, and its predictive performance was assessed. (3) Results: The model had an area under the curve (AUC) of 0.95, with a true positive rate of 86.7%, and a false discovery rate of 10.5%. The overall accuracy of our model was 90%. (4) Conclusion: This is the first study in the literature that evaluated the performance of a FFBPN for the prediction of pregnant patients with thrombophilia at a high risk of giving birth to SGA newborns, and its promising results could lead to a tailored prenatal management.

## 1. Introduction

Fetal growth restriction (FGR) is a condition characterized by the fetal inability to grow to its expected biological potential in utero. Pre- and postnatal consequences of fetal growth restriction include: stillbirths, neonatal hypoglycemia, hypocalcemia, polycythemia, and respiratory depression [1]. In the current literature, the definitions of FGR, intrauterine growth restriction (IUGR), and small for gestational age (SGA) fetuses are heterogenous [2]. Moreover, the terms are used interchangeably for both the pre- and postnatal period, but for the purpose of this article, we have chosen the SGA definition that proposes a neonatal weight < 10th percentile according to population charts.

Depending on the FGR definition used, it is estimated that between 3% and 9% of pregnancies in the developed world, and up to 25% of pregnancies in low- and middle-income countries, are affected by this condition [3,4].

Fetal growth depends on maternal factors (including maternal health status, nutritional status, smoking, drug use, etc.), fetal factors (genetic background), and placental function [5]. One of the most important maternal disorders associated with intrauterine growth restriction is thrombophilia, regardless of its etiology (inherited or acquired) [6,7,8].

It has been postulated that thrombophilic mutations negatively influence the utero-placental blood flow and induce fetal vasculopathy, thus ultimately determining poor fetal growth [9,10,11]. A meta-analysis by Hemsworth et al. revealed a 40% increase in the risk of intrauterine growth restriction (IUGR) in factor V Leyden mutation carriers [12]. Several retrospective studies outlined the associations between IUGR and antithrombin III deficiency [13], PAI-I [6], MTHFR gene polymorphisms [14], etc. A recent meta-analysis and systematic review outlined the possible association between MTHFR 677C > T polymorphism and the development of IUGR [15].

Current research evaluated numerous algorithms for the prediction of intrauterine growth restriction that used maternal characteristics, serum biomarkers (PLGF, sFLT-1, etc.), and sonographic parameters, some of them having good accuracy, especially for late-onset fetal growth restriction (≥32 weeks of gestation) [16,17,18,19,20]. Despite good predictive value, their implementation is difficult in developing countries where the patient’s compliance to a rigorous monitoring program is low, and the financial possibilities are limited.

More recently, artificial neural networks (ANN) and machine learning (ML) techniques have emerged as innovative options for adverse pregnancy outcomes prediction, allowing complex analysis of numerous clinical and paraclinical parameters [21,22,23]. One of the most used machine learning techniques for developing predictive models is the Feed-Forward Back Propagation Network (FFBPN), which describes the complex relationship between the input and output values of a network set [24]. An input layer of neurons, a hidden layer of neurons, and an output layer of neurons make up a standard multi-layer feed forward neural network. Every node in a layer is connected to another node in the adjacent forward layer [25]. The back-propagation algorithm employs a gradient descent rule, which aims to reduce the network’s error by moving down the gradient of the error curve, resulting in a good overall accuracy for the FFBPN [26].

The aim of this study was to evaluate the predictive performance of a FFBPN that comprises clinical and paraclinical parameters for the prediction of small for gestational age newborns in a cohort of pregnant patients with thrombophilia. This is the first study in the current literature that explores the predictive performance of this method for the specified pathology.

## 2. Materials and Methods

We conducted an observational retrospective study of all pregnancies that occurred in women with thrombophilia who attended two tertiary maternity hospitals: ‘Cuza-Voda’, Iasi, and ‘Saint John Emergency Hospital’, Suceava, Romania, between January 2013 and December 2020. Ethical approval for this study was obtained from the Institutional Ethics Committees of University of Medicine and Pharmacy ‘Grigore T. Popa’ (No. 17806/03.09.2019), ‘Cuza-Voda’ Maternity Hospital, Iasi (No. 1254/01.02.2022) and ‘Saint John Emergency Hospital’, Suceava (No. 7.21.01.2022). Informed consent was obtained from all participants included in the study. All methods were carried out in accordance with relevant guidelines and regulations.

Medical records of patients were systematically reviewed and data obtained. The inclusion criteria taken into consideration were: pregnant patients with thrombophilia and maternal age ≥ 18, singleton pregnancies with first trimester pregnancy dating, and a SGA diagnosis.

Exclusion criteria comprised patients who had multiple pregnancies, ectopic pregnancies, first and second trimester abortions, fetal intrauterine demise, fetuses with chromosomal or structural abnormalities, intrauterine infection, incomplete medical records, incorrect/lack of first trimester sonographic pregnancy dating, or who were unable to offer informed consent due to various reasons (age less than 18 years old, intellectual deficits, psychiatric disorders, etc.).

The following variables were recorded: demographic data, the patient’s medical history, BMI (body mass index), smoking status during pregnancy, laboratory parameters (the presence of factor V Leiden, MTHFR A1298C homozygosity, MTHFR C677T homozygosity, plasminogen activator inhibitor 1 deficiency (PAI-1) deficiency, antithrombin III deficiency, protein S and C deficiency, resistance to activated protein C (APCR), prothrombin, lupus anticoagulant (LAC), and anticardiolipin (ACL) IgM and IgG antibodies), and pregnancy outcome.

All pregnant women were evaluated by an experienced obstetrician with an early ultrasound scan using an E8/E10 (General Electric Healthcare, Zipf, Austria) scanner with a 4.8 MHz transabdominal probe (GE Medical Systems, Milwaukee, WI) between 10 + 0 and 13 + 6 weeks to determine gestational age by measuring the crown–rump length (CRL) [27]. The patients were followed over the entire course of their pregnancies, and were segregated into two groups: no SGA (group 1) and with SGA (group 2). In this paper, the terms SGA, IUGR, and FGR are used interchangeably. SGA was defined as the birth of a neonate with birthweight < 10th centile according to Lubchenco Growth Curves for term infants and Fenton Growth Charts for premature infants [28,29].

Statistical analysis was performed using SPSS software (version 28.0.1, IBM Corpora-tion, Armonk, NY, USA). Bivariate associations of APO status and each predictor variable were evaluated with chi-square and Fisher’s exact tests for categorical variables and T-test for continuous variables. A *p*-Value less than 0.05 was considered statistically significant.

**Figure 1:**
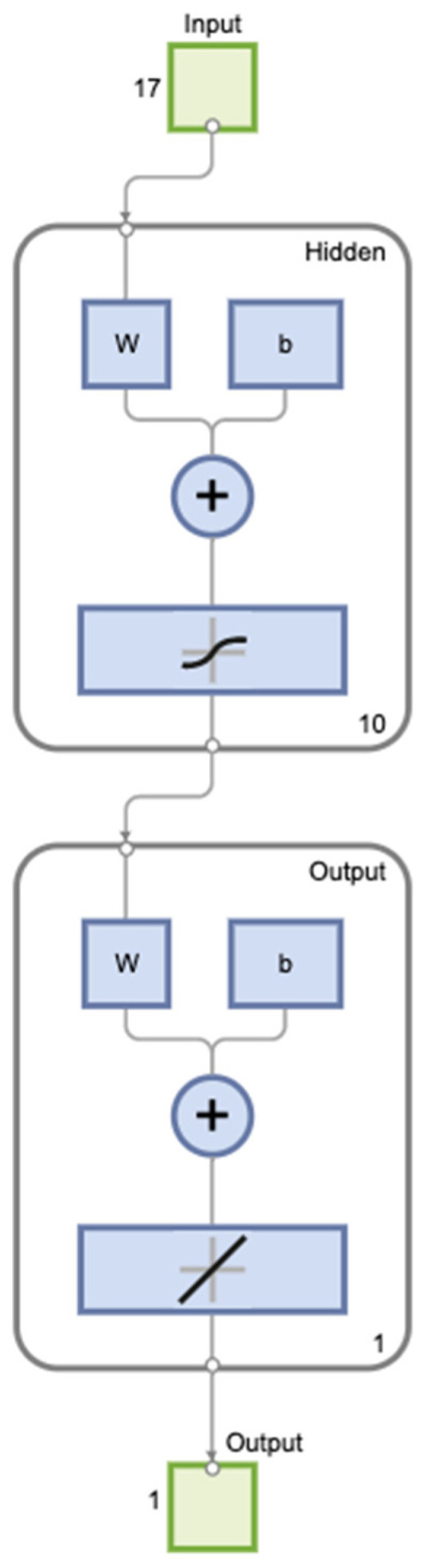
Graphic representation of the FFBPN model.

The final dataset, comprised of 466 cases, was loaded and checked to see whether it had any missing values or not. If any missing values were found, they were replaced to a null value. Variables with a significant *p*-Value from the univariate analysis, as well as paraclinical data without statistical significance, were entered into a FFBPN as input variables, along with the presence of SGA as an output variable using Matlab (version R2021b). We performed the features scaling using the standardization method. The input and output layers comprised 17 and 1 neuron, respectively, corresponding to the numbers of predictors and output variable (Figure 1) [30,31]. The optimal number of neurons in the hidden layer (H) was determined from the prediction model with the highest sensitivity and specificity, and our hidden layer consisted of 10 neurons. The original data were split into training data (80%) and test data (20%).

The training parameters were set at their default values. The Levenberg–Marquardt algorithm was used as the training function. Continuous log-sigmoid functions were used as the transfer functions of the hidden and output layers. The network was trained at a maximum of 10 epochs. Confusion matrix and ROC curve were used for determining the predictive value of the artificial neural network.

## 3. Results

The first step of the study was to identify predictive factors of growth restriction from a combination of clinical and laboratory data. Our results showed that age (*p* = 0.021), a high maternal BMI (*p* < 0.001), current smoking (*p* < 0.001), chronic maternal hypertension (*p* < 0.001), and a personal history of ischemic placental disease (preeclampsia, intrauterine growth restriction, and abruptio placentae) (*p* < 0.001) were clinical parameters with significant statistical influence over the occurrence of SGA in current pregnancy (Table 1).

On the other hand, Factor V Leiden (*p* < 0.001), MTHFR A1298C homozygous (*p* < 0.001), MTHFR C677T homozygous (*p* < 0.001), PAI-I deficiency (*p* < 0.001), and AT III deficiency (*p* < 0.001) were thrombophilia mutations with significant impact over the SGA occurrence later in pregnancy (Table 1).

**Figure 2:**
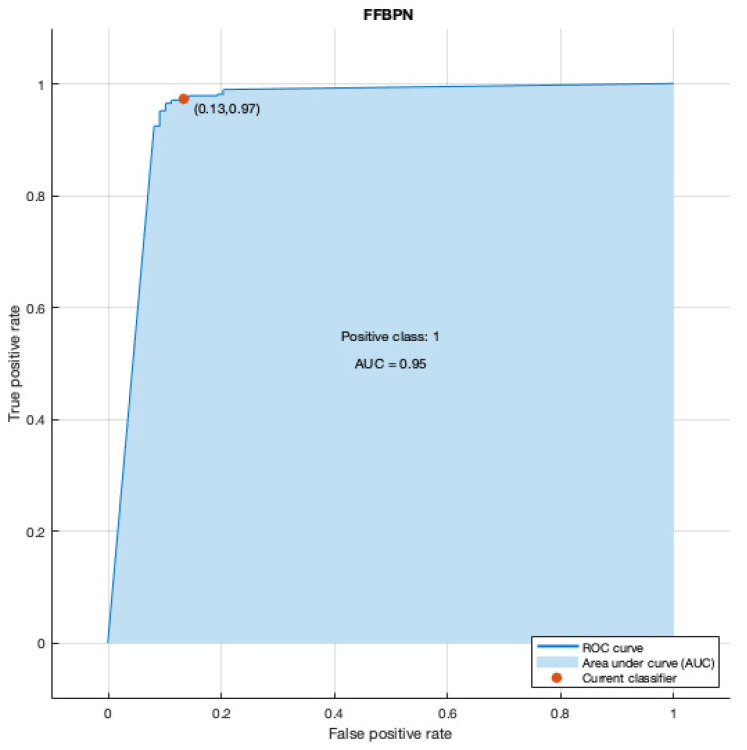
Graphic representation of area under the curve (AUC) and receiver operating characteristic curve (ROC) for our Feed-Forward Back Propagation Network (FFBPN).

The second step was to construct a prediction model using FFBPN for prediction in our cohort of patients with thrombophilia. Our network consisted of three layers and had an area under the curve (AUC) of 0.95 (Figure 2).

**Figure 3:**
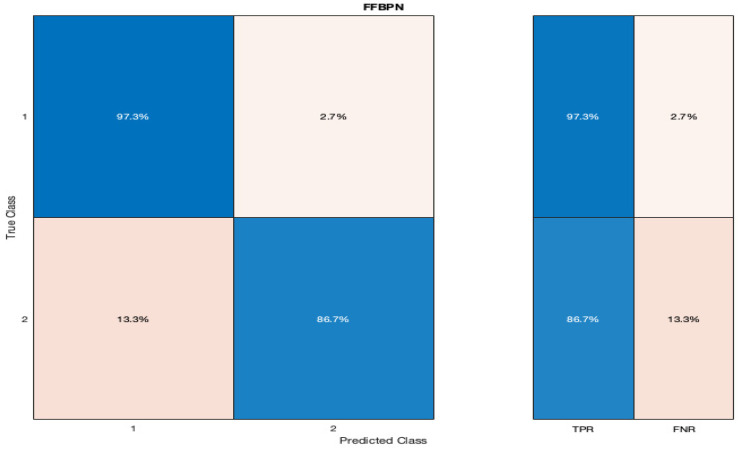
Graphic representation of the confusion matrix with the true positive rates (TPR) and false negative rates (FNR) for the prediction of SGA newborns (listed here as 2) when using a Feed-Forward Back Propagation Network (FFBPN).

**Figure 4:**
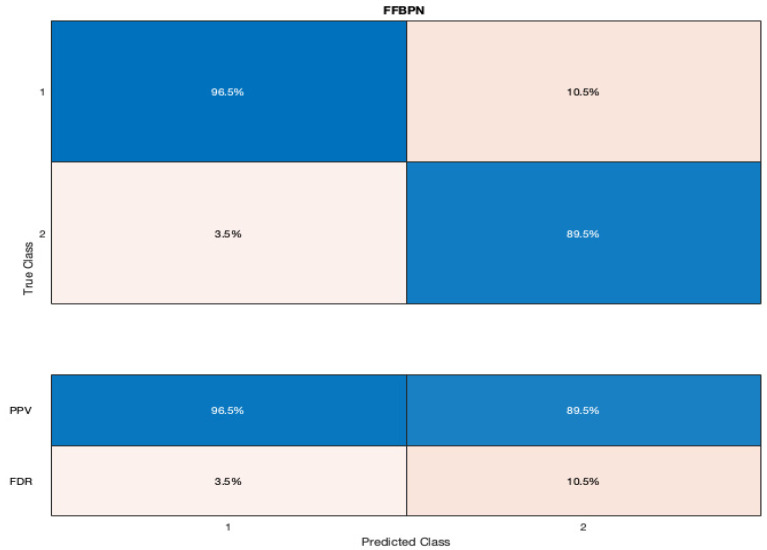
Graphic representation of the confusion matrix with the positive predictive values (PPV) and false discovery rates (FDR) for the prediction of SGA newborns (listed here as 2) when using a Feed-Forward Back Propagation Network (FFBPN).

The confusion matrices for our model are represented in Figure 3 and Figure 4. The true positive rate (TPR) of our FFBPN for the prediction of SGA was 86.7%, while the false negative rate (FNR) was 13.3%. The positive predictive value (PPV) for the SGA detection was 89.5%, and the false discovery rate (FDR) was 10.5% when using our artificial neural network. The precision, recall, and F1 score for the SGA prediction were 0.89, 0.86, and 0.88, respectively. The accuracy of our model was 90%.

**Figure 5:**
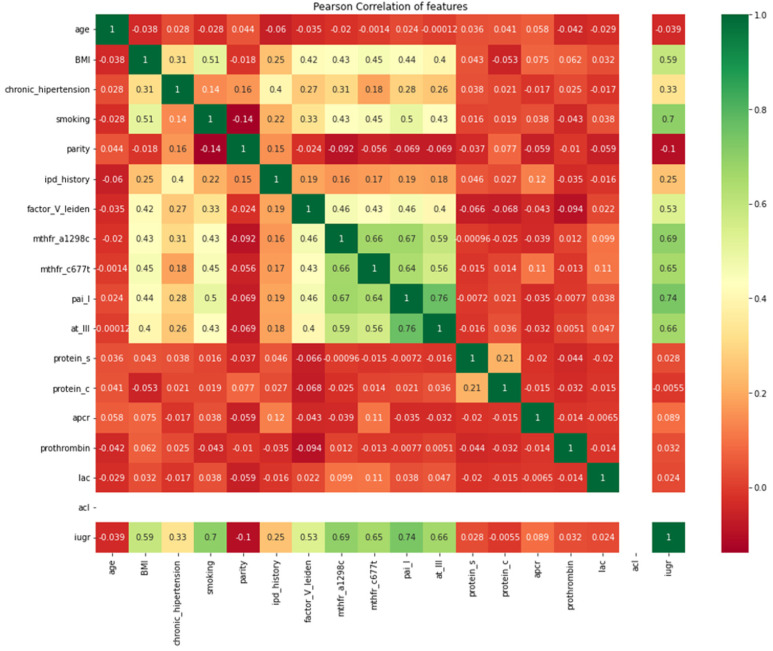
Graphic representation of the Pearson correlation matrix for the included predictors.

Finally, we measured, using a Pearson correlation matrix, the strength and direction of a linear relationship between the predictor variables (Figure 5).

Our results showed a positive correlation between SGA newborns and PAI-I deficiency (*r* = 0.74), current smoking status (*r* = 0.7), MTHFR A1298C homozygous (*r* = 0.69), MTHFR C677T homozygous (*r* = 0.65), antithrombin III (*r* = 0.66), and factor V Leiden (*r* = 0.53).

## 4. Discussion

In this observational retrospective study, we trained a Feed-Forward Back Propagation Network on 466 pregnant patients with thrombophilia to predict small for gestational age newborns. The model had an area under the curve of 0.95, with a true positive rate of 86.7% and a false discovery rate of 10.5% for SGA detection. The F1 score, which is used to assess the quality of the model with values ranging from 0 to 1, was 0.88. This result, along with an accuracy of 90%, indicate a good predictive performance of the model.

As far as we know, this is the first study that evaluated the predictive performance of FFBPN for the prediction of SGA neonates in a particular cohort of pregnant patients diagnosed with thrombophilia. However, this method has been implemented in several studies for the prediction of various disorders such as gastric cancer (accuracy of 92.27%) [32], gastroduodenal ulcer (accuracy of 90%) [33], lung cancer (accuracy of 96%) [34], etc. Our prediction results are comparable with findings from other studies. Thus, this type of neural network could be used in further studies in order to predict various obstetrical outcomes.

Many of the existing models for predicting adverse pregnancy outcomes are risk score models that are based on epidemiological data and/or clinical factors. Singh et al. developed a weighted risk score model for the prediction of low birth weight using six variables (weight gain in the mother during pregnancy, intake of proteins in diet, history of preterm birth, history of low birth weight, maternal anemia, and passive smoking), and obtained a sensitivity of 72% [35]. In this study, we established a FFBPN for SGA prediction using comprehensive maternal clinical and paraclinical data and achieved a sensitivity of 86.7%. The superiority and reasonableness of BPNN models in solving complex nonlinear interactions is demonstrated by our prediction model, which surpasses the above -mentioned method.

Our results showed a positive correlation between SGA newborns and inherited thrombophilia mutations such as PAI-I deficiency, MTHFR A1298C homozygous, MTHFR C677T homozygous, antithrombin III, and factor V Leiden (*r* = 0.53), and at the same time a negative correlation with acquired thrombophilia markers such as lupus anticoagulant (*r* = 0.024). These data suggest a higher impact of inherited thrombophilia mutations over the occurrence of growth restriction later in pregnancy for our cohort of patients. Indeed, several studies outlined the association between inherited thrombophilia mutations and IUGR [12,14,36], but only limited data supported the association between acquired thrombophilia mutations and IUGR [7], thus further studies are needed to evaluate the impact of these thrombophilic mutations over the pregnancy’s outcomes.

Our study has several limitations, including a small cohort of patients and number of predictors, but at the same time, the proposed neural network has the advantage of an easier implementation by the physicians.

We hypothesize that the model’s accuracy could be improved by adding maternal serum biomarkers such as PLGF, sFlt-1, and their ratios, as well as sonographic parameters such as cerebro-placental index, fetal abdominal circumference, and the pulsatility index for uterine arteries. Nonetheless, this model could be externally validated on larger cohorts of patients and, depending on the results, could be used for the prediction of small for gestational age neonates in a cohort of pregnant patients with thrombophilia. Our model has the advantage of using easily accessible maternal characteristics, as well as routinely screened thrombophilia mutations for patients with a high-risk profile, and can be applied as soon as the first trimester of pregnancy.

The identification of pregnant patients with thrombophilia who are at high risk of developing growth restriction during gestation since the first trimester is a key element for adopting a prevention strategy based on low-dose aspirin and/or low molecular weight heparin. It was hypothesized that administration of low molecular weight heparin since the first trimester of pregnancy could limit the occurrence of ischemic placental disease in patients with thrombophilia [37,38], but further research is needed to elucidate the exact mechanism of action of these drugs.

Although the implementation of artificial neural networks and deep learning algorithms for disease prediction is still an emerging field of medical research, we are hopeful that the development of new techniques will allow better patient classification and individualized management.

## 5. Conclusions

Despite the limitations, this is the first study that evaluated the predictive performance of a FFBPN which comprised clinical and paraclinical parameters for the prediction of small for gestational age newborns in a cohort of pregnant patients with thrombophilia. Using the FFBPN model, we can identify pregnant women at high-risk of SGA in early pregnancy, and the results could be helpful for obstetricians in guiding prenatal management.

## Figures and Tables

**Table 1:** Characteristics of pregnant patients with thrombophilia and their association with intrauterine growth restriction (IUGR).

Patient Data		No SGA	SGA	*p*-Value
Demographics	Age	Mean = 31.99 ± 4.11 SD	Mean = 31.51 ± 4.96 SD	0.021
	BMI	Mean= 22.7 ± 21.56 SD	Mean= 25.42 ± 1.2 SD	<0.001
Patient’s history	Parity	Primiparity Yes = 212 (74.9%)	Primiparity Yes = 71 (25.1%)	0.054
	Smoking	Yes = 10 (13.2%) No = 358 (91.8%)	Yes = 66 (86.8%) No = 32 (8.2%)	<0.001
	Chronic hypertension	Yes = 3 (15%) No = 365 (81.8%)	Yes = 17 (85%) No = 81 (18.2%)	<0.001
	History of ischemic placental disease	Yes = 5 (26.3%) No = 363 (81.2%)	Yes = 14 (73.7%) No = 84 (18.8%)	<0.001
Paraclinical data	Factor V Leiden	Yes = 36 (36%) No = 332 (90.7%)	Yes = 64 (64%) No = 34 (9.3%)	<0.001
	MTHFR A1298C homozygous	Yes = 17 (19.5%) No = 351 (92.9%)	Yes = 70 (80.5%) No = 27(7.1%)	<0.001
	MTHFR C677T homozygous	Yes = 15 (19%) No = 353 (91.5%)	Yes = 64 (81%) No = 33 (8.5%)	<0.001
	PAI I deficiency	Yes = 7 (9.5%) No = 361 (92.1%)	Yes = 67 (90.5%) No = 31 (7.9%)	<0.001
	AT III deficiency	Yes = 6 (9.5%) No = 362 (89.8%)	Yes = 57 (90.5%) No = 41 (10.2%)	<0.001
	PROTEIN S deficiency	Yes = 20 (74.1%) No = 348 (79.3%)	Yes = 7 (25.9%) No = 91 (20.7%)	0.520
	PROTEIN C deficiency	Yes = 12 (80%) No = 356 (78.9%)	Yes = 3 (20%) No = 95 (21.1%)	0.921
	APCR	Yes = 1 (33.3%) No = 367 (79.3%)	Yes = 2 (66.7%) No = 96 (20.7%)	0.052
	Prothrombin	Yes = 10 (71.4%) No = 358 (79.2%)	Yes = 4 (28.6%) No = 94 (20.8%)	0.482
	LAC	Yes = 2 (66.7%) No = 366 (79%)	Yes = 1 (33.3%) No = 97 (21%)	0.600
	ACL	Yes = 2 (66.7%) No = 366 (79%)	Yes = 1 (33.3%) No = 97 (21%)	0.600

## Data Availability

The data presented in this study are available on request from the corresponding author. The data are not publicly available due to local policies.
